# Relations Between Core Taxa and Metabolic Characteristics of Bacterial Communities in *Litopenaeus vannamei* Ponds and Their Probiotic Potential

**DOI:** 10.3390/microorganisms13020466

**Published:** 2025-02-19

**Authors:** Qiong Zhao, Ke Zhou, Fengfeng Zhang, Yu Wang, Jun Hao, Fengxing Xie, Qian Yang

**Affiliations:** 1 Tianjin Institute of Agriculture Resource and Environment, Tianjin Academy of Agricultural Science, Tianjin 300384, China; zhaoqiong000@126.com (Q.Z.); zhoukeisrael@sina.com (K.Z.); izff@163.com (F.Z.); 2 Tianjin Fisheries Research Institute, Tianjin 300221, China; 13602055400@163.com (Y.W.); jhao402042774@163.com (J.H.); 3 Harbin Institute of Technology, School of Life Science and Technology, Harbin 150001, China

**Keywords:** aquatic probiotics, bacterial community, keystone and core taxa, carbon source utilization, nitrogen degradation, *Tropicimonas*

## Abstract

Microorganisms play a crucial role in purifying aquaculture water bodies. However, there is limited understanding regarding the core species of bacterial communities in aquaculture ponds and their metabolic functions. Using 16S rRNA gene sequencing technology, network analysis, and Biolog EcoPlates, we identified keystone and core taxa of bacterial communities in *Litopenaeus vannamei* ponds and investigated their correlations with their community’s carbon source utilization abilities based on Biolog EcoPlates. We found that keystone and core taxa in bacterial communities were significantly correlated with the carbon source utilization abilities of bacterial communities. The positively correlated core taxa include (1) *Bacillus*, *Flavobacterium*, *Brevibacillus*, and *Paenibacillus*, which are used as probiotics in aquaculture, and (2) *Candidatus* Aquiluna, *Dechloromonas*, *Sulfurifustis*, *Terrimicrobium*, *Alsobacter*, and *Gemmobacter*, which have been reported to play a role in nitrogen removal. Furthermore, the positively correlated *Tropicimonas* (Rhodobacterales: Rhodobacteraceae) in aquaculture has not yet been applied. By nitrogen degradation experiments in aquaculture wastewater, we confirmed the synergistic relationship between the genera *Tropicimonas* and *Bacillus*. The co-introduction of *Tropicimonas sediminicola* SDUM182003 and *Priestia aryabhattai* HG1802 or *Bacillus subtilis* XQ1804 into the aquaculture tailwater reduced the time required for the removal rates of nitrite nitrogen and nitrate nitrogen to reach over 90% by 24–48 h. Our research reveals the correlation between core taxa and community carbon source utilization, indicating that the core taxa of bacterial communities play a crucial role in the metabolic functions of the community, and offering a reference for exploring new bacterial genera with probiotic potential.

## 1. Introduction

In recent years, global aquaculture production has been steadily increasing, with a record high production of 130.9 million tons in 2022, thereby exceeding the yield of capture fisheries for the first time and emerging as the primary source of aquatic animal products [1]. However, aquaculture also faces several environmental challenges, including pollution of the surrounding water body due to improper discharge of wastewater and waste products during the cultivation process. Aquatic probiotics are extensively used in aquaculture to purify water bodies and prevent diseases; however, the microbial genera in the existing aquatic probiotics have remained somewhat the same, with few reported new bacterial genera. Current research indicates that the keystone taxa influence the structure and function of the microbial community; thus, it is anticipated that these species also play a crucial role in the metabolic processes of the community. Recent studies have indicated that aquatic probiotics facilitate nutrient cycling in ponds by upregulating the metabolic functions of microbial communities [2] (Li et al., 2022). Given that aquatic microbial species regulate community metabolic functions, they might also be one of the core species within these communities. The simultaneous identification of core species significantly associated with community metabolic functions in aquaculture ponds could serve as a novel approach to determine the appropriate microbial genera for application as aquatic probiotics.

The advent of high-throughput sequencing technology has facilitated in-depth and extensive investigations into bacterial communities residing in shrimp ponds [3,4]. Recently, microbial network analysis, based on high-throughput sequencing, has been widely used to analyze the evolution of bacterial communities, as well as to identify keystone taxa that drive community structure and function [5,6]. Current studies have shown that the core microbial species in the sediments of shrimp ponds primarily include a few groups, and the relative abundance of these groups is closely associated with the physical and chemical properties of the sediments [7]. Network analysis revealed that *Candidatus* Aquiluna, a member of the actinomycete family, is a crucial species in intensive *Litopenaeus vannamei* aquaculture ponds and potentially functions as a nitrogen-removing bacterial species [8].

Keystone taxa are highly connected taxa that exert a considerable influence on microbiome structure and functioning relative to their abundance. In interspecific networks, these species have high mean degree, low betweenness centrality, and high closeness centrality [9]. As such, values of within-module connectivity (*Zi*) and among-module connectivity (*Pi*) can be used to identify keystone taxa in a community [10]. The k-core decomposition, which identifies the largest subgraph of a network [11], can also be used to identify core taxa in microbial communities [12,13]. The size of the k-core value indicates that in the core subnetwork, each node is connected to at least k other nodes [11]. The score of the k-core decomposition reflects the density of the seed node and surrounding node; the larger the score, the better stability and higher degree of irreplaceability and importance of the genus in the entire microbial community [14].

While network analysis can be used to identify potential interactions among different taxa in a community based on their co-occurrences or antagonistic associations [15], it cannot explain the ecological functions of microorganisms in a microbial network. Therefore, it is necessary to integrate network analysis with information on the physiological and metabolic traits of microbial species and to elucidate how different species, especially keystone and core taxa, contribute to community-level metabolism.

Biolog EcoPlates technology is a method primarily used in the study of community-level physiological profiling (CLPP) [16]. The technology measures the functional diversity of bacterial communities based on the different types of carbon substrates used by microorganisms [17], thereby facilitating links between community structure and actual metabolic function [18]. Therefore, the present study aimed to reveal the keystone and core microbial taxa in the water and sediments of shrimp ponds and elucidate the correlation of these key microbial species with the utilization of grouped carbon sources of bacterial communities based on Biolog EcoPlates. By identifying key microbial species with significant positive correlations that have not yet been widely applied, we evaluated the potential of individual key microbial species for application as aquatic probiotics based on nitrogen degradation experiments in aquaculture tailwater.

We focused on ponds used for the intensive aquaculture of the shrimp *L. vannamei*. We constructed the molecular ecological networks of the bacterial communities in the water and sediments of the ponds and identified keystone and core bacterial taxa. Simultaneously, we used Biolog EcoPlates to conduct a correlation analysis of the keystone and core taxa of the bacterial communities and the capacity of bacterial community to metabolize different carbon sources. Finally, we assessed the functionality of the taxa with probiotic potential for nitrogen degradation in aquaculture tailwaters. The following three hypotheses were proposed: (1) a significant correlation exists between the keystone taxa in aquaculture ponds and the carbon source utilization ability of pond bacterial communities based on Biolog EcoPlates; (2) the microbial genera currently used in aquatic probiotics might be comparable with the core species in aquaculture ponds; and (3) core species showing a significant positive correlation with the carbon source utilization capacity of pond microbial communities could be applied as aquatic probiotics. The study of the aforementioned hypothesis can provide evidence to confirm the significant role of core taxa in community metabolic functions, offer new insights into investigating the mechanism of action of aquatic probiotics at the community level, and also present novel approaches for discovering new genera of aquatic probiotics.

## 2. Materials and Methods

### 2.1. Water and Sediment Samples

The ponds were located at the breeding base of the Animal Husbandry and Aquaculture Bureau, Xiqing District, Tianjin, China. The stocking density of shrimp larvae was 600,000 per hectare, and 5 kg of commercial shrimp feed was fed into each acre of pond twice daily during the cultivation period. Shrimp were successfully cultured in the three ponds, with no major disease outbreak.

Water samples WS1, WS2, and WS3 and sediment samples SD1, SD2, and SD3 were collected from three adjacent shrimp culture ponds (0.5 ha) in the later stage of shrimp farming. Due to the increase in food and waste in the pond, it is particularly important to evaluate the relationship between the core taxa in shrimp-intensive aquaculture ponds and their metabolic functions during the later stage of aquaculture.

Surface water samples (0.2–0.5 m below the water surface) were collected using an organic glass water sampler. Surface sediment samples (0–5 cm depth) were collected using a cylindrical sampler. All samples were collected from the central area and four corners of each pond and mixed to form a composite biological replicate sample, with five replicates established for each sample. All samples were transported in sterile sampling bottles, stored at 4 °C, and then immediately sent to the laboratory for further experiments.

### 2.2. CLPP Analysis Using Biolog EcoPlates

An eight-channel pipette was used to add the sample solution to the preheated Biolog EcoPlates at the rate of 150 μL per well. The sediment suspension used for inoculating microplate wells was prepared as follows: (1) fresh sediment equivalent to 10 g of dry sediment was accurately weighed and added to sterile physiological saline containing glass beads to prepare a 10% sediment suspension; (2) the suspension was shaken at 250 rpm for 30 min, and 30 mL of the sediment suspension was transferred to a 50 mL centrifuge tube; (3) the suspension was centrifuged at 600 rpm for 10 min, and the supernatant was directly transferred to Biolog EcoPlates; (4) the plates were covered and placed in a freshness preservation box, and the box was incubated at 25 °C; and (5) the absorbance value of each well was read at 590 and 750 nm wavelengths every 24 h by using a Biotek ELx808 microplate reader. Three water samples from each pond were added to the Biolog EcoPlates. Each plate had three replicates, with a total of nine replicates [19]. Data were collected from the sediment samples after incubation for 216 h [20] and from water samples after incubation for 48 h [21].

The 31 distinct carbon sources present in the Biolog EcoPlate are categorized into six groups: 6 amino acids, 12 carbohydrates, 5 carboxylic acids, 4 polymers, 2 amines, and 2 phenolic acids [22]. The average OD_590_–OD_750_ value for each carbon source category was calculated to determine the utilization of these sources by the bacterial community.

### 2.3. DNA Extraction, Bacterial 16S rRNA Gene Amplification, and Illumina Sequencing Analysis

To analyze microbial community structure, WS1-WS3 were initially filtered through a sterilized 5 μm filter membrane, and the filtrate was transferred into a sterile bottle. Subsequently, the bacterial cells in the filtrate were decanted onto a 50 mm diameter filter membrane with a 0.22 μm pore size using a filtration apparatus, and the membrane was placed into a 5 mL cryogenic vial. The sediment and filter membrane samples collected were stored at −80 °C in a refrigerator for future use.

The pretreated water and sediment samples were transported to Tianjin Novogene Biological Information Technology Co., Ltd. at −20 °C for microbial community analysis. Primer 515F (5′-GTGCCAGCGGTAA-3′) -806R (5′-GACTACHVGGGTWTCTAAT-3′) was selected for amplicon sequencing in the 16S V4 region. The specific procedures for DNA extraction, the construction of sequencing libraries, the sequencing methods, and Illumina sequencing analysis have been described by Zhao et al. [21]. The raw sequencing data obtained from this study were submitted to the National Center for Biotechnology Information Sequence Read Archive (SRA) and BioProject under ID PRJNA544379.

### 2.4. Network Analysis and Identification of Potential Keystone Taxa and Core Taxa

We accessed the iNAP online analysis platform (https://inap.denglab.org.cn/) to construct and analyze the bacterial network on 7 July 2023 [23]. We then used Gephi software (version number is 0.10.1) to visualize the bacterial network [24]. Data on the absolute abundances of bacteria at the genus level in the water and sediment samples were used as the input files for these analyses. First, the bacterial genera in the water and sediment samples were filtered such that only genera that were present in more than half of all samples in each category were retained in the analysis. Next, the SparCC method was used to calculate the correlation coefficient and *p*-value. The correlation threshold of the bacterial communities in the water and sediment was determined to be 0.69 using the random matrix theory (cutoff) method [25]. The significance of the difference was determined to be *p* < 0.05. Nine topological parameters were calculated to evaluate the interaction between the bacterial communities within the two networks.

We classified the nodes of each network into different roles according to their within-module connectivity (*Zi*) and among-module connectivity (*Pi*) values, as follows: module hubs (*Zi* ≥ 2.5, *Pi* < 0.62), network hubs (*Zi* ≥ 2.5, *Pi* ≥ 0.62), connectors (*Zi* < 2.5, *Pi* ≥ 0.62), and peripherals (*Zi* < 2.5, *Pi* < 0.62) [10]. We considered taxa that were module hubs, network hubs, and connectors as potential keystone taxa due to their important roles in network topology. The calculations of *Zi* and *Pi* were performed on the iNAP. We used the K-core decomposition method in Gephi software to identify core taxa in the largest core subset of each network.

### 2.5. Application of Tropicimonas in Nitrogen Degradation Experiments

Strains of *Tropicimonas sediminicola* were obtained from Shandong Research Center of Marine Microbiological Culture Collection and Application Engineering Technology, No. SDUM182003,and were isolated from the culture pond. To verify the nitrogen removal efficiency of *T. sediminicola* SDUM182003 in aquaculture, it was compared with a strain of *Priestia aryabhattai* HG1802 (collection number: CGMCC No. 29019) and *Bacillus subtilis* XQ1804, which were isolated and screened in our laboratory and could efficiently degrade nitrite. These three strains were inoculated separately into Zobell 2216E medium and simulated seawater culture medium supplemented with 25 mg/L sodium nitrate to determine their nitrogen removal efficiency in sterile culture solutions. The composition of the simulated seawater culture medium was as follows: 0.3 g/L of beef extract, 0.5 g/L of sucrose, 35 g/L of sea salt, 0.075 g/L of KH_2_PO_4_, pH 7.6.

To validate the synergistic effect between *Tropicimonas* and *Bacillus*, we inoculated the strains of *T. sediminicola* SDUM182003, *P. aryabhattai* HG1802, and *B. subtilis* XQ1804 in the culture tailwater of *Amphiprion ocellaris* supplemented with ammonia nitrogen (NH_3_-N) and nitrite nitrogen (NO_2_-N). Initially, the activated strains of *T. sediminicola* SDUM182003, *P. aryabhattai* HG1802, and *B. subtilis* XQ1804 were transferred, respectively, to a conical flask and grown under shaking culture, which was followed by bacterial cell collection. The cells were washed with sterile physiological saline, and a bacterial suspension of the three strains was prepared at the concentration of 1.2 × 10^9^ CFU/mL (as determined by McFarland’s turbidimetry). Subsequently, 900 mL of the culture tailwater of *A. ocellaris* was added to a round aquaculture tank and supplemented with 0.03 g/L (NH_4_)_2_SO_4_, 0.5 g/L sucrose, 0.075 g/L KH_2_PO_4_, and 25 mg/L NaNO_2_. The water body contained 8.23 mg/L NH_3_-N, 5.24 mg/L NO_2_-N, and 11.86 mg/L nitrate nitrogen (NO_3_-N).

*T. sediminicola* SDUM182003 and *P. aryabhattai HG1802* were then fed into the round culture tank either individually or in a 1:1 volume ratio. The concentration of introduced bacteria in the tank was 2.7 × 10^6^ CFU/mL. A tank containing no inoculation was designated as a control. Each tank was placed in an incubator maintained at 37 °C. After 24 to 96 h of culturing, the treated water was collected and centrifuged at 8000 r/min for 10 min. The resulting supernatant was analyzed to determine the levels of NH_3_-N, NO_2_-N, and NO_3_-N, and then the removal rates of both were calculated. The measurements of ammonia (NH_3_-N), nitrite (NO_2_-N), and nitrate (NO_3_-N) were conducted following the methods outlined in Xie et al. [26]. The mixed culture method was used for *T. sediminicola* SDUM182003 and *B. subtilis* XQ1804 as described above. Following nutrient addition, the effluent contained 11.89 mg/L NH_3_-N, 4.18 mg/L NO_2_-N, and 14.07 mg/L NO_3_-N.

### 2.6. Statistical Analysis

Statistical analyses were primarily performed using SPSS version 19.0 for Windows (IBM Corp., Armonk, NY, USA, 2010). Data on keystone taxa abundance, physicochemical indicators, and carbon source utilization by bacterial community were subjected to outlier removal by using the local outlier factor method available at the Majorbio Cloud Platform (https://cloud.majorbio.com/page/tools/) on 16 July 2024. After the outliers were removed from the physicochemical factors, the mean value was used for imputation. Subsequently, the physicochemical factors for redundancy analysis were determined by SPSS collinearity diagnosis and the Akaike information criterion. Correlation analyses were conducted using the “psych” package [27] and visualized using the “pheatmap” package [28] in RStudio software (Version 1.1.383) [29]. Redundancy analysis (RDA) was performed between keystone species and pond water environmental factors by using CANOCO 5.0 (Microcomputer Power, Ithaca, NY, USA). Differences in the NH_3_-N, NO_2_-N, and NO_3_-N removal efficiency between samples were analyzed using a one-way ANOVA. *p* values of *p* < 0.05 were considered statistically significant.

## 3. Results

### 3.1. Bacterial Community Network at the Genus Level

A total of 574 genera were annotated from the bacterial communities in water and sediment samples. In water samples, the dominant genera with relative abundance greater than 1% included unidentified *Cyanobacteria*, unidentified *Synechococcales*, *Polynucleobacter*, unidentified *Alphaproteobacteria*, unidentified *Acidimicrobiia*, *Candidatus* Aquiluna, *Acidibacter*, *Lautropia*, *Thiobacillus*, unidentified *Balneolaceae*, unidentified *Nostocaceae*, and *Acinetobacter*. In the sediment samples, the dominant genera with relative abundance greater than 1% included *Thiobacillus*, *Smithella*, *Methanolinea*, *Methanosaeta*, and *Syntrophirhabdus*.

The co-occurrence patterns of bacterial communities in the water of the shrimp ponds were found to be more complex than those in the sediment (Table 1). Specifically, the number of nodes, the number of edges, average degree, and average clustering coefficient of the co-occurrence network of bacterial communities in the water were 99, 782, 15.798, and 0.679, respectively, while these were 115, 398, 6.922, and 0.551, respectively, in the co-occurrence network of the bacterial communities in the sediment. The higher average clustering coefficient, average degree, and number of edges in the co-occurrence network for bacterial communities in water were indicative of their stronger ecological connections. At the same time, the proportions of positive associations in the co-occurrence networks of bacterial communities in the water and sediment were 57.8% and 60.8%, respectively. This indicates that bacterial communities in pond environments display a greater degree of cooperation than competition.

**Figure 1:**
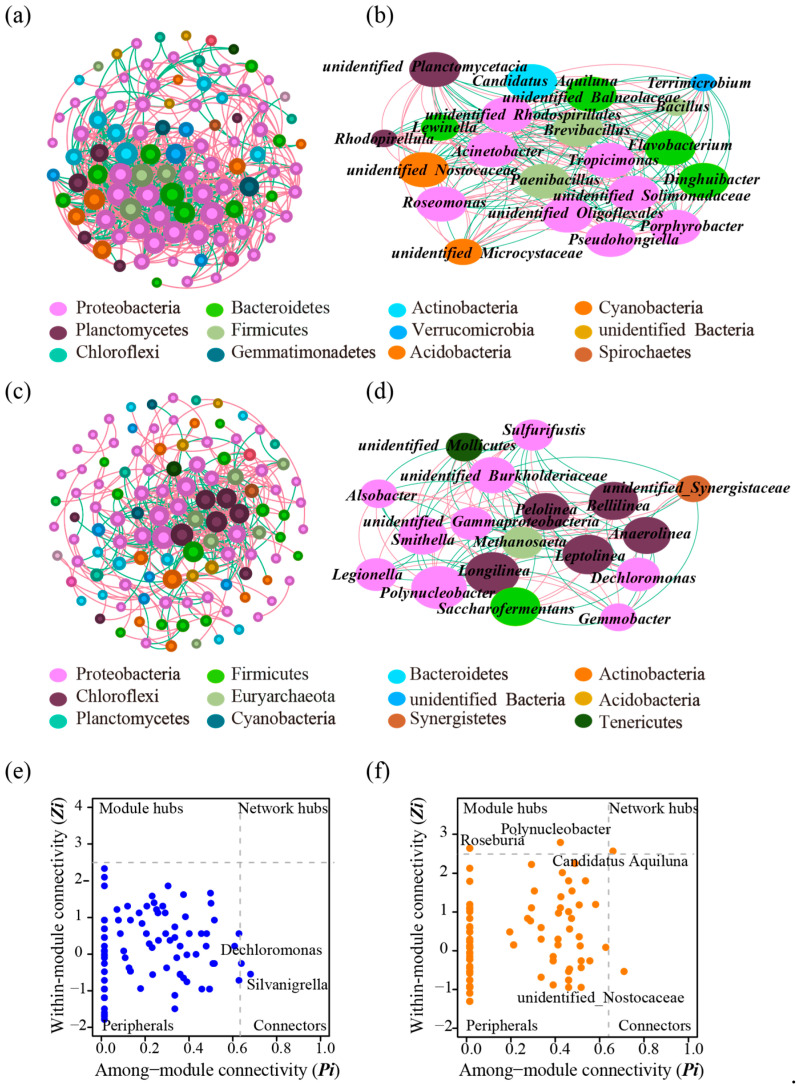
Co-occurrence network analysis of bacterial communities in water and sediment. (**a**): The genus-level co-occurrence network of the bacterial community in the water body. (**b**): The largest k-18 subset in the co-occurrence network of the bacterial community in the water body, which comprised 21 core genera. (**c**): The genus-level co-occurrence network of the bacterial community in the sediment. (**d**): The largest k-9 subset in the co-occurrence network of the bacterial community in the sediment, which comprised 18 core genera. The size of each node is proportional to the degrees. The red and green lines indicate positive and negative correlations, respectively. (**e**,**f**): Identification of keystone taxa in the water body (**e**) and the sediment (**f**), based on their topological roles in networks.

Based on their relative abundances, the dominant phyla in the co-occurrence network of the bacterial communities in the water were Proteobacteria (49.49%), Bacteroidetes (10.1%), Actinobacteria (8.08%), Cyanobacteria (6.06%), Planctomycetes (5.05%), Verrucomicrobia (4.04%), Firmicutes (3.03%), as well as unidentified Bacteria (3.03%) and Chloroflexi (3.03%) (Figure 1a). Among these phyla, Proteobacteria displayed the most interactions with other bacteria, with relative abundances of 49.49% in the co-occurrence networks of communities in the water.

The k-core decomposition is to find the largest subgraph of a network, in which each node has at least k neighbors in the subgraph. In the network of the bacterial community in the water, the largest k-core value was 18; the genera involved in this core subnetwork included genera of *Flavobacterium*, *Acinetobacter*, *Brevibacillus*, *Tropicimonas*, *Candidatus* Aquiluna, *Porphyrobacter*, unidentified *Rhodospirillales*, *Paenibacillus*, unidentified *Solimonaceae*, *Pseudohongiella*, *Dinghuibacter*, unidentified *Nostocaceae*, unidentified *Oligoflexales*, *Terrimicrobium*, unidentified *Balneolaceae*, *Lewinella*, *Roseomonas*, *Bacillus*, *Rhodopirellula*, unidentified *Microcystaceae*, and unidentified *Planctomycetacia* (Figure 1b).

The dominant phyla in the co-occurrence network of the bacterial community in the sediment were Proteobacteria (43.48%), Firmicutes (10.43%), Actinobacteria (6.96%), Bacteroidetes (6.96%), Chloroflexi (6.09%), Euryarchaeota (5.52%), unidentified Bacteria (4.35%), and Acidobacteria (3.48%) (Figure 1c). Proteobacteria also displayed the most interactions with other bacteria, with relative abundances of 43.48% in the co-occurrence networks of communities in the sediment.

In the network of the bacterial community in the sediment, the largest k-core value was 9; the genera involved in this core subnetwork included genera of *Polynucleobacter*, *Longilinea*, *Leptolinea*, *Saccharofermentans*, *Bellilinea*, *Smithella*, *Anaerolina*, *Pelolinea*, unidentified *Burkholderiaceae*, *Dechloromonas*, *Legionella*, unidentified *Gammaproteobacteria*, *Methanosaeta*, *Sulfurifustis*, *Alsobacter*, unidentified *Mollicutes*, *Gemmobacter*, and unidentified *Synergistaceae* (Figure 1d).

Two genera, belonging to the genera *Dechloromonas* and *Silvanigrella*, were identified as connectors in the network of the bacterial community in the water based on their topological roles (Figure 1e). In the network of the bacterial community in the sediment, two module hubs, one connector, and one network hub were identified (Figure 1f); these were genus of *Polynucleobacter*, *Roseburia*, unidentified *Nostocaceae*, and *Candidatus* Aquiluna, respectively.

### 3.2. Correlation Analyses of the Relationships Between Keystone and Core Taxa and the Utilization of Grouped Carbon Sources in Bacterial Communities

**Figure 2:**
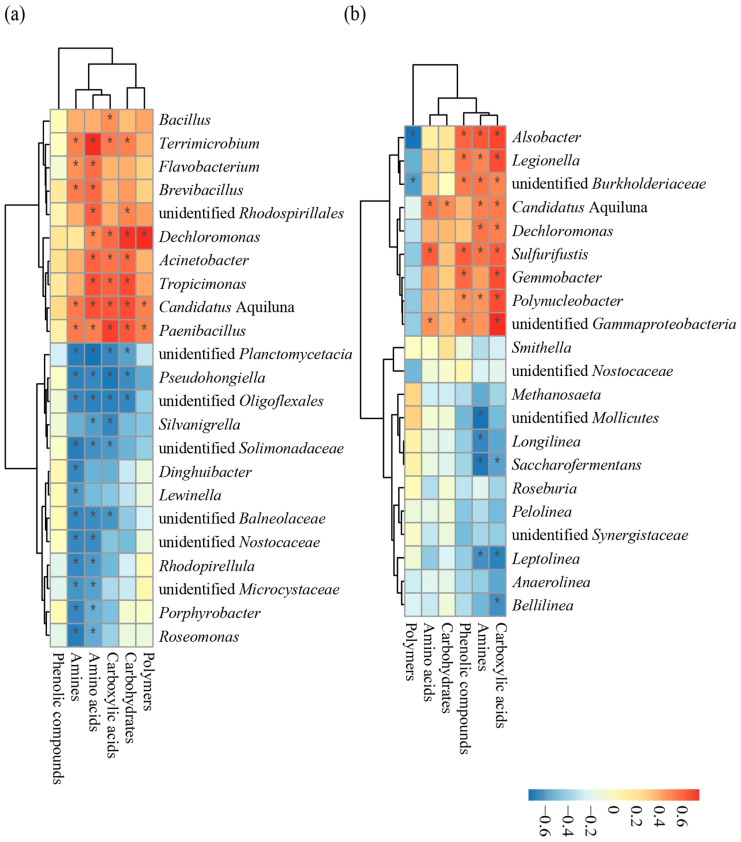
Correlations between the utilization of grouped carbon sources at the community level and the keystone and core taxa of bacterial communities in the water body (**a**) and sediment (**b**). The color intensity shows the *R*-value of correlation in each panel, and the asterisk represents significant correlations at *p* < 0.05 level. Note: data on keystone taxa abundance and carbon source utilization by bacterial community were subjected to outlier removal.

The keystone and core taxa of the bacterial community in the water were significantly correlated with the utilization of carbon sources at the community level, based on the Biolog EcoPlates analysis (Figure 2). Among these, the keystone taxa and core taxa which showed significant positive correlations with the community-level utilization of carbon sources included *Dechloromonas*, *Candidatus* Aquiluna, *Paenibacillus*, *Bacillus*, *Tropicimonas*, *Brevibacillus*, *Acinetobacter*, unidentified *Rhodospirillales*, *Flavobacterium*, and *Terrimicrobium.* The keystone and core taxa displayed significant negative correlations with the community-level utilization of carbon sources included unidentified *Planctomycetacia*, *Pseudohongiella*, unidentified *Balneolaceae*, unidentified *Oligoflexales* unidentified *Nostocaceae*, unidentified *Solimonadaceae*, *Rhodopirellula*, and *Silvanigrella* etc.

In addition to *Roseburia*, unidentified *Nostocaceae*, *Anaerollinea*, *Smithella*, unidentified *Synergistaceae*, *Methanosaeta*, and *Pelolinea*, the keystone and core taxa of the bacterial community in the sediment were significantly correlated with the utilization of carbon sources at the community level. Here, the core taxa that showed a significant positive correlation with the community-level utilization of carbon sources included *Sulfurifustis*, unidentified *Gammaproteobacteria*, *Candidatus* Aquiluna, *Gemmobacter*, *Legionella*, *Polynucleobacter*, *Dechloromona*, and other bacterial genera, while taxa that were significantly negatively correlated with the community-level utilization of carbon sources included *Leptolinea*, *Longilinea*, *Saccharofermentans*, unidentified *Mollicutes*, and *Bellilinea*.

### 3.3. Correlations Between Keystone and Core Taxa of Bacterial Communities and Nitrogen Indices in the Water Samples

**Figure 3:**
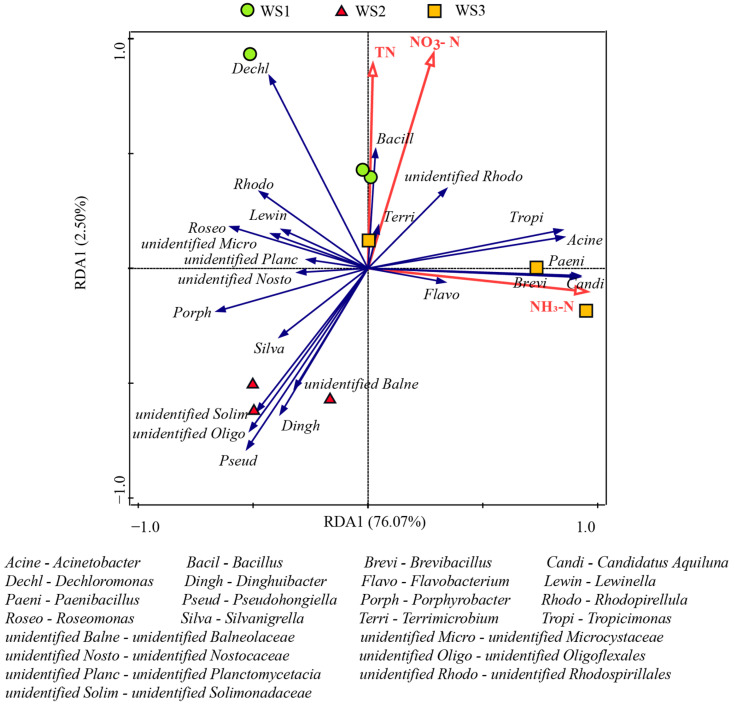
Redundancy analysis (RDA) showing the relationship between TN, NH_3_-N, and NO_3_-N and the keystone and core taxa of bacterial communities in water samples.

The nitrogen indices of water samples in shrimp ponds have been described in our previously published article [30]. Table S1 shows the physical and chemical characteristics of the water samples and sediment samples collected from the shrimp ponds. TN, NH_3_-N, and NO_3_-N were chosen as the variables in the RDA model to explain the variations in the keystone and core taxa of bacterial communities in the water samples. NH_3_-N showed a significant correlation with the structural changes in the keystone and core taxa of bacterial communities (*p* = 0.014), with explanation rates of 69.3% (Figure 3). As shown in Figure 3, the core taxa of bacterial communities in the water samples, such as *Candidatus* Aquiluna, *Paenibacillus*, *Bacillus*, *Tropicimonas*, *Brevibacillus*, *Acinetobacter*, *unidentified Rhodospirillales*, *Flavobacterium*, and *Terrimicrobium*, were positively correlated with changes in the concentrations of NH_3_-N and NO_3_-N. In addition to *Dechloromonas*, *Dechloromonas* is positively correlated with the utilization of NO_3_-N and negatively correlated with the utilization of NH_3_-N. This result was generally consistent with the observations based on the Biolog EcoPlates.

### 3.4. Application of T. sediminicola in Nitrogen Degradation Experiments

**Figure 4:**
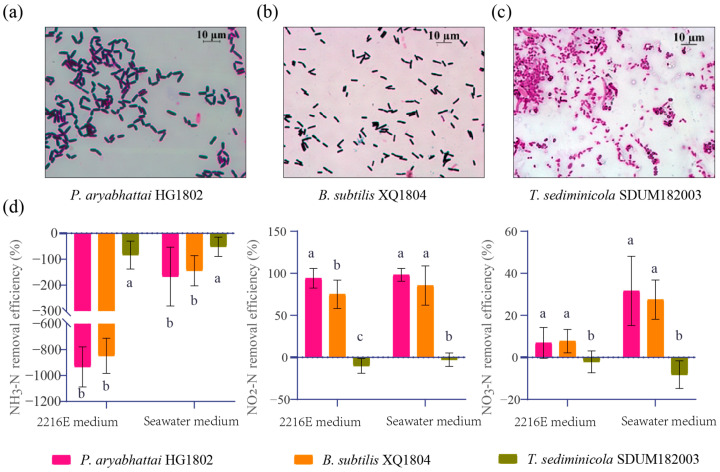
Gram staining images of *P. aryabhattai* HG1802 (**a**), *B. subtilis* XQ1804 (**b**), and *T. sediminicola* SDUM182003 (**c**) and nitrogen removal efficiency of these three strains in 2216E culture medium and simulated seawater culture medium for 24 h (**d**). In subgraph d, the lowercase letters a, b, and c denote the significant differences (*p* < 0.05) in the removal efficiency of NH_3_-N, NO_2_-N, and NO_3_-N among the three strains cultured in the same medium.

The microbial community structure of the shrimp aquaculture was analyzed, and the metabolic functions of the bacterial community were assessed by Biolog EcoPlates. The results revealed that the genus *Tropicimonas* showed a significant positive correlation with the metabolic function of the bacterial community and a positive correlation with NH_3_-N concentration in the aquaculture water. Notably, to date, there are no reports on the application of this genus in aquaculture. To investigate the effectiveness of nitrogen removal by *Tropicimonas*, the following were added to the aquaculture tailwater: *T. sediminicola* SDUM182003, *B. subtilis* XQ1804, and *P. aryabhatai* HG1802. Figure 4a–c show Gram staining images of these strains. *B. subtilis* XQ1804 and *P. aryabhattai* HG1802 exhibited notable NO_2_-N removal efficiency in 2216E culture medium and simulated seawater culture medium, whereas *T. sediminicola* did not show nitrogen removal capability in both culture media (Figure 4d).

A correlation was observed between *Tropicimonas* and various core bacterial genera in the analysis of the bacterial community network in the water samples (Table 2). We found significant positive correlations between *Tropicimonas* and three other genera: *Brevibacillus* (*r =* 0.98), *Paenibacillus* (*r* = 0.95), and *Bacillus* (*r* = 0.87). To assess the synergistic relationship between *Tropicimonas* and *Bacillus*, *T. sediminicola* SDUM182003, *P. aryabhattai* HG1802, and *B. subtilis* XQ1804 were added to the aquaculture wastewater to determine their nitrogen removal efficiency.

**Figure 5:**
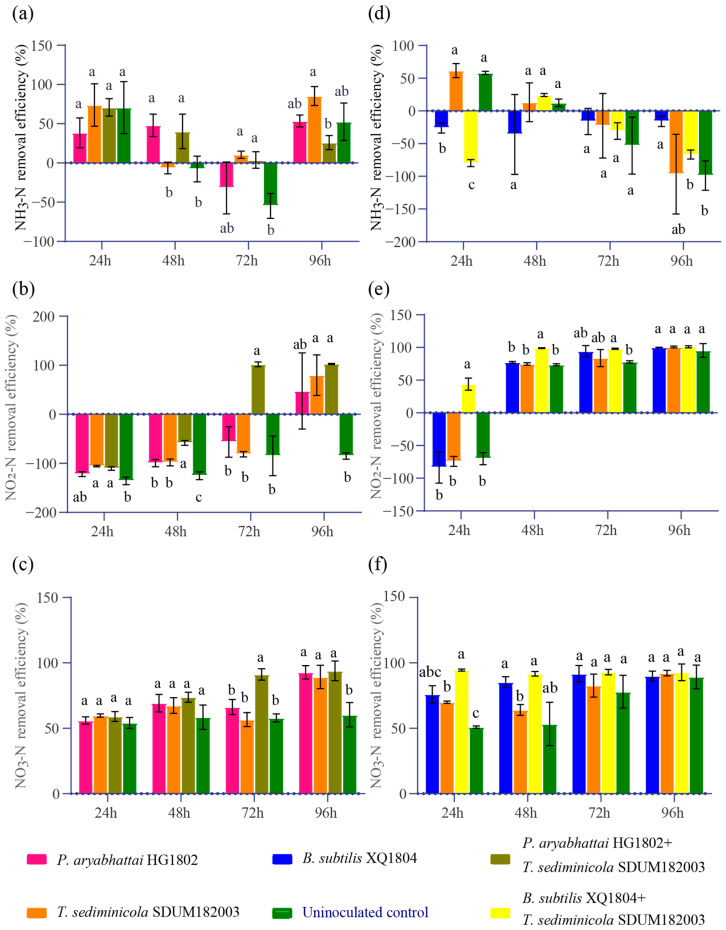
NH_3_-N, NO_2_-N, and NO_3_-N removal efficiency in the aquaculture tailwater based on the interaction between *T. sediminicola* SDUM182003 and *P. aryabhattai* HG1802 (**a**–**c**), and *T. sediminicola* SDUM182003 and *B. subtilis* XQ1804 (**d**–**f**). The lowercase letters in the figure indicate the significance of differences among various treatments at a given time point, with treatments sharing the same letter indicating non-significant differences (*p* < 0.05).

The addition of a *T. sediminicola* SDUM182003 and *P. aryabhattai* HG1802 combination and of a *T. sediminicola* SDUM182003 and *B. subtilis* XQ1804 combination in the natural aquaculture wastewater with diverse microbial communities yielded distinct patterns of nitrogen transformation as compared to their addition to sterile culture media. The combined inoculation of *T. sediminicola* SDUM182003 and *P. aryabhattai* HG1802 slightly altered NH_3_-N levels in the wastewater, with a reduction in both NO_2_-N and NO_3_-N to their lowest levels within the shortest time period of 72 h (Figure 5a–c). The introduction of the *T. sediminicola* SDUM182003 and *B. subtilis* XQ1804 combination into the aquaculture wastewater initially significantly increased NH_3_-N levels and later showed no notable differences in NH_3_-N levels when compared with those obtained after the addition of *T. sediminicola* SDUM182003 alone or uninoculated control. However, the introduction of *T. sediminicola* SDUM182003 and *B. subtilis* XQ1804 in combination significantly decreased the levels of NO_2_-N and NO_3_-N in the aquaculture wastewater within 24 h (Figure 5d–f).

## 4. Discussion

### 4.1. Correlations Between Keystone and Core Taxa and Carbon Source Utilization Capacity at the Community Level

The core species of microbial communities are considered regulators of the structure and function of the overall community. Microbial communities play an important role in the material cycle of aquaculture water through their metabolic functions. Thus, it is expected that the core microbial species of aquaculture ponds will remarkably affect the metabolic characteristics of the pond microbial community. Previous studies have shown a significant correlation between the composition and metabolic characteristics of microbial communities in water bodies [18,30]. On the basis of this viewpoint, we conducted experiments to assess the relationship between core species and community carbon and nitrogen metabolism. The results showed that, except for some bacterial genera in the sediment community, the key core bacterial species in the shrimp pond water and sediment microbial communities were significantly associated with the carbon and nitrogen metabolism capacity of the community.

A reason for this finding is that the keystone and core taxa in the community constitute the cornerstone of the entire network and may interact with other bacterial species through mutualisms and competition. Metabolic cross-feeding appears to be the main driver of successional dynamics [31], and the metabolic division of labor is a common form of microbial interaction and community metabolic strategy [32]. Therefore, based on their substrate-utilizing capabilities, keystone and core taxa in the microbial community may facilitate cross-feeding interactions among various species, thereby influencing the structure and function of the microbial community. Research on soil microbiomes has reported significant correlations between the activities of keystone species and the utilization of organic matter [5,33]. This information may help to elucidate the community assembly mechanisms of bacterial communities that involve their metabolic functions.

As mentioned earlier, *Roseburia*, unidentified *Nostocaceae*, *Anaerollinea*, *Smithella*, unidentified *Synergistaceae*, *Methanosaeta*, and *Pelolinea* did not show significant correlations with the community-level utilization of six types of carbon sources in the sediment; this could be due to the fact that the Biolog EcoPlates conduct aerobic cultivation. The genera of *Roseburia*, *Anaerolinea*, *Smithella*, *Methanosaeta*, and *Pelolinea* are mostly distributed in anaerobic environments. Future studies should thus use the Biolog AN Plates to explore the correlations of keystone and core taxa of bacterial communities in sediment with carbon source utilization at the community level in anaerobic environments.

### 4.2. Relationship Between the Core Species in Shrimp Pond Water and the Major Aquatic Probiotics Currently in Use

To determine the relationship between the core species in shrimp ponds and the currently used aquatic probiotics, we grouped the core species based on the quantity and types of carbon source utilized by them. In aquatic microbial communities, 10 core taxonomic groups exhibited a significant positive correlation with the utilization of six types of carbon sources by the community.

*Candidatus* Aquiluna and *Paenibacillus* exhibit a significant positive correlation with the utilization of five types of carbon sources, excluding phenolic compounds. *Candidatus* Aquiluna, a lineage of actinomycetes, has been reported to be a keystone taxon of bacterioplankton communities in the intensive cultivation of *Litopenaeus vannamei* [34]. Recent studies suggest that this species may also contribute to nitrogen removal [35]. *Paenibacillus polymyxa* has been used in shrimp farming and was found to promote nutrient absorption and enhance shrimp immunity by regulating the composition of intestinal microbiota [36].

*Dechloromonas* is significantly positively correlated with the utilization of four carbon sources other than amines and phenolic compounds. *Dechloromonas* belongs to the γ- Gammaproteobacteria subfamily Rhodocyclaceae. Studies have found that *Dechloromonas* can simultaneously remove nitrogen and phosphorus in anoxic environments through the denitrification phosphorus removal metabolic pathway, thereby reducing demands for sources of carbon and aerobic activity [37]. *Terrimicrobium* is significantly positively correlated with the utilization of carbon sources other than phenolic compounds and polymers. *Terrimicrobium*, a strictly anaerobic bacteria, was found to be the dominant genera in a lab-scale anoxic–oxic moving bed biofilm reactor (A/O-MBBR) used to treat simulated nitrogenous tailwater [38].

*Tropicimonas* and *Acinetobacter* exhibit a significant positive correlation with the utilization of carbohydrates, carboxylic acid, and amino acids as carbon sources. *Tropicimonas* (Rhodobacterales: Rhodobacteraceae) are mostly isolated from marine environments [39,40]. *Acinetobacter*, belonging to the class of Gammaproteobacteria in the order of Pseudomonadales, is a non-fermenting, Gram-negative bacterium widely found in nature and is a conditionally pathogenic bacterium. Unidentified *Rhodospirillales* is significantly positively correlated with the utilization of carbohydrates and amino acids as carbon sources. Some of the photosynthetic bacteria widely used in aquaculture currently belong to the Rhodospirillales order.

*Flavobacterium* and *Brevibacillus* are significantly positively correlated with the utilization of amino acids and amine carbon sources. *Flavobacterium* has been identified as a probiotic, although it may cause fish diseases in some instances [41]. Research has shown that the addition of *Flavobacterium* BA-3 or its extracellular products to feed can promote the innate immunity of fish to disease [42]. *Brevibacillus laterosporus* plays a role in dissolving algae in aquaculture water by secreting antibiotics and proteases [43].

*Bacillus* species are extensively used in agricultural production and aquaculture. As a key species in aquatic microbial communities, *Bacillus* species primarily utilize carboxylic acid as a carbon source. Thus, the diverse carbon sources that showed a significant correlation with each abovementioned bacterial genus are likely to be associated with the broad range of carbon source utilization of these genera and their specific preferences within the aquaculture environment.

Nine core taxonomic groups within the sediment microbial community exhibit a significant positive correlation with the community’s utilization of diverse carbon sources. *Candidatus* Aquiluna is significantly and positively correlated with the utilization of four carbon sources, excluding phenolic compounds and polymers, whereas *Sulfurifusis* shows a significant positive correlation with the utilization of carbon sources, excluding polymers and carbohydrates. *Sulfurifustis* is an important contributor to both sulfur oxidation and denitrification in mangrove sediments [44].

*Alsobacter*, *Legionella*, unidentified *Burkholderiaceae*, and *Polynucleobacter* are significantly positively correlated with the utilization of carboxylic acid, amines, and phenolic compounds as carbon sources by sediment microbial communities. *Alsobacter ponti*, a new species of the genus *Alsobacter*, isolated from the sediments of Zhuhai, has potential to perform denitrification and sulfur reduction [45]. *Legionella* is widely distributed in all natural aquatic environments, moist soil, and marshlands, and its abundance remarkably increases in the guts of rapidly growing shrimp [46]. *Polynucleobacter*, belonging to the Burkholderiaceae family in the Gammaproteobacteria class, exhibits two ecological types: free-living and symbiotic, and it is widely distributed across freshwater ecosystems [47]. Although *Polynucleobacter* has seldom been used in aquaculture probiotics thus far, a few studies have found that their abundances in aquaculture systems can be significantly increased by the addition of *Bacillus* [48].

Unidentified *Gammaproteobacteria* is significantly positively correlated with the utilization of amino acid, phenolic, and carboxylic acid carbon sources; *Dechloromona* is significantly positively correlated with carbon source utilization of carboxylic acids and amines; *Gemmobacter* is significantly positively correlated with the utilization of carboxylic acid and phenolic carbon sources. *Gemmobacter denitrificans* sp. nov. is a denitrifying bacterium that can remove nitrites and nitrates from pond water used for culturing *L. vannamei* [49].

In summary, among the strains showing a significant positive correlation with the metabolic characteristics of shrimp pond water and sediment microbial communities, the genus *Bacillus* and the order Rhodospirillales, which include various photosynthetic bacteria, are frequently used as probiotics in aquaculture, while *Flavobacterium*, *Brevibacillus*, and *Paenibacillus* have the potential for use as aquatic probiotics [50,51]. As mentioned earlier, *Candidatus* Aquiluna, *Dechloromonas*, *Sulfurifustis*, *Terrimicrobium*, *Alsobacter*, and *Gemmobacter* also play a role in nitrogen removal from shrimp pond water. These results indicate a certain overlap between the core species showing a positive correlation with the microbial community grown on Biolog EcoPlates and the currently widely used aquatic probiotics. The analysis also revealed that the core species with a crucial role in community function should have good carbon and nitrogen metabolism capabilities, whereas most existing aquatic microbial strains are primarily selected according to their nitrogen removal capacity. This reveals the reason for the certain overlap between the core species of aquatic microbial communities and the reported aquaculture probiotic. This indicates that positive correlations between the microbial community composition and its metabolic characteristics based on Biolog EcoPlates may provide a tool to identify novel aquatic probiotics. However, whether the core species of the pond bacterial community achieve cross-feeding through effective metabolic functions, thereby exhibiting stronger ecological connections, and the interrelationships between each core bacterial genus, still require further research.

### 4.3. Application of Tropicimonas to Aquaculture Tailwater

In analyzing the structure of the microbial community in a shrimp aquaculture and examining the metabolic functions of the bacteria via Biolog EcoPlates, we found that the genus *Tropicimonas* was significantly positively correlated with the carbon and nitrogen metabolism pathways of the bacterial community. Notably, there have been no reports of this genus from marine environments being used in aquaculture to date. However, the Rhodobacteriaceae family, which includes *Tropicimonas*, constitutes a major core microbiota in the intestines of Pacific white shrimp (*Penaeus vannamei*) and is considered a potential probiotic candidate for improving shrimp farming [52].

To investigate the probiotic potential of *Tropicimonas* species, we treated the aquaculture tailwater of the marine ornamental fish *A. ocellaris* with *T. sediminicola* SDUM182003. *T. sediminicola* SDUM182003 showed significantly lower nitrogen removal efficiency when cultured in 2216E liquid medium and simulated seawater culture medium. However, after its co-introduction with *P. aryabhattai* HG1802 or *B. subtilis* XQ1804 into the aquaculture tailwater, it reduced the time required for the degradation rates of NO_2_-N and NO_3_-N in the aquaculture water to reach over 90% by 24–48 h.

*B. subtilis* is commonly used as a water quality improver for aquaculture water. *Bacillus aryabhattai*, a novel species in the genus *Bacillus*, was reported in 2009. In 2020, 17 branches of the genus *Bacillus* were recognized as new genera, and *Bacillus aryabhattai* was subsequently renamed *Priestia aryabhattai* [53]. Currently, *P. aryabhattai* is considered a promising candidate probiotic and can function as an antagonistic species for pathogens, particularly in aquaculture [54]. Current transcriptomic studies have elucidated the mechanism through which Mg^2+^ ions enhance nitrite degradation in aquaculture wastewater by *B. aryabhattai* 47 [55].

The present study, based on network analysis and nitrogen degradation experiments, revealed that *T. sediminicola* SDUM182003 and *B. subtilis* or *P. aryabhattai* act synergistically to remove nitrogen from aquaculture wastewater, wherein *T. sediminicola* enhances the nitrogen degradation capabilities of *B. subtilis* and *P. aryabhattai*. Synergism among multiple microbial strains may enable them to better thrive in complex and constantly changing environments. *Rhodobacter* and *Tropicimonas* are members of the Rhodobacteraceae family, and Rhodobacter has a diverse range of metabolic capabilities, including photosynthesis, lithotrophy, and aerobic and anaerobic respiration. These species can also fix nitrogen. These capabilities allow *Rhodobacter* to survive in various habitats [56]. Moreover, a member of the Rhodobacteraceae family promotes initial biofilm formation by secreting extracellular factor(s) [57]. Therefore, it is hypothesized that the diverse metabolic capabilities of *Tropicimonas*, along with the potential secretion of specific extracellular substances by this species, may enhance the water purification function of *Bacillus*. Hence, the specific synergistic mechanisms between *Tropicimonas* and *Bacillus* require further investigations.

Although the core species are considered to have a probiotic role based on their strong influence on community structure and function, in the present study, we experimentally confirmed for the first time that the core species can also function as a water purification agent. However, because the performance of a single bacterial strain cannot replace that of a genus, further studies are required with other *Tropicimonas* strains in various aquaculture settings. It should also be noted that the nitrogen removal performance of these bacterial species varied significantly between sterilized pure culture media and wastewater containing a diverse range of microorganisms, thus highlighting the need to investigate not only the functions of individual bacterial strains in pure cultures but also the impact of aquatic probiotics on the physicochemical parameters and microbial communities in aquaculture water bodies. It is also crucial to determine the mechanism of action of aquatic probiotics at the community level and to explore novel aquatic probiotics.

## 5. Conclusions

In this study, we found that keystone and core taxa in bacterial communities in the water and sediment of shrimp ponds were significantly correlated with the carbon source utilization abilities of their respective communities. Our findings indicate that the keystone and core taxa of the community may play a role in community assembly through their substrate utilization ability. Our study also identified keystone taxa and core taxa that positively correlated with carbon source utilization ability include the main genera currently used in aquatic probiotics, as well as other genera with the potential for use as novel probiotics. By performing nitrogen degradation experiments in aquaculture wastewater, we confirmed the presence of a synergistic relationship between the genera *Tropicimonas* and *Bacillus*. Although the robust influence of core species on the structure and function of the overall microbial community suggests their potential probiotic role, the present study experimentally confirmed for the first time that core species with a significant positive correlation with community metabolic function in aquaculture ponds can also serve as a water purification agent. This indicates that positive correlations between the composition of the microbial community and its metabolic characteristics observed on Biolog EcoPlates may provide a strategy to identify novel aquatic probiotics. However, due to the ongoing need for optimization of identification methods for core species in microbial communities and the application of Biolog EcoPlates across various fields, coupled with the limited range of carbon sources available on Biolog EcoPlates, there are certain limitations in exploring probiotics based on core community species and metabolic characteristics. Additionally, the isolation and verification of probiotics discovered through this process present certain challenges. Further studies are required in the future to explore the application of this method across different fields to enable the discovery of various functional strains.

## Figures and Tables

**Table 1:** Topological properties of the molecular ecological networks of bacterial communities at the genus level in water samples and sediment.

Topological Properties	Water Samples	Sediment
Number of nodes	99	115
Number of edges	782	398
Network density	0.161	0.061
Average degree	15.798	6.922
Network diameter	6	9
Positive/negative association (%)	57.8/42.2	60.8/39.2
Average path length	2.457	3.388
Average clustering coefficient	0.679	0.551
Modularity	0.318	0.440

**Table 2:** Correlation between *Tropicimonas* and various core bacterial genera of the bacterial community network in water from three adjacent shrimp culture ponds.

Genus	Interaction	Correlation
*Brevibacillus*	positive	0.9849
*Paenibacillus*	positive	0.9547
*unidentified_Rhodospirillales*	positive	0.9529
*Flavobacterium*	positive	0.8802
*Bacillus*	positive	0.8721
*Rhodopirellula*	negative	−0.7601
*unidentified_Microcystaceae*	negative	−0.7736
*unidentified_Planctomycetacia*	negative	−0.7772
*unidentified_Oligoflexales*	negative	−0.8079
*Pseudohongiella*	negative	−0.8147
*Roseomonas*	negative	−0.8355
*unidentified_Solimonadaceae*	negative	−0.8476
*Dinghuibacter*	negative	−0.8594
*Porphyrobacter*	negative	−0.9075

## Data Availability

The data underlying this article are available in Science Data Bank at https://doi.org/10.57760/sciencedb.11772. The raw sequencing data obtained from this study were submitted to the National Center for Biotechnology Information Sequence Read Archive (SRA) and BioProject under the following ID: PRJNA544379.

## Supplementary Materials

The following supporting information can be downloaded at: https://www.mdpi.com/article/10.3390/microorganisms13020466/s1, Table S1: Physical and chemical characteristics of water samples (WS1–WS3).
