# Supplementary material for: Relations Between Core Taxa and Metabolic Characteristics of Bacterial Communities in Litopenaeus vannamei Ponds and Their Probiotic Potential
Source: Microorganisms. 2025 Feb 19;13(2):466. doi: 10.3390/microorganisms13020466 (PMC11858629; doi:10.3390/microorganisms13020466)
Supplement: Supplementary file 1 [file microorganisms-13-00466-s001.zip › microorganisms-3409524-supplementary.pdf]

Table S1. Physical and chemical characteristics of water samples (WS1–WS3).

and sediment samples (SD1–SD3) from three adjacent shrimp culture ponds

	WS1	WS2	WS3	SD1	SD2	SD3
Organic content (%)	—	—	—	23.24%	18.64%	15.38%
BOD	7.3	6.8	9.0	—	—	—
DO（mg/L）	7.90	8.64	8.69			
Temperature (℃)	26	26	27			
pH	8.26	8.23	8.26			

BOD: biochemical oxygen demand, DO: dissolved oxygen.
